# Supplementary material for: Previously undetected super-spreading of Mycobacterium tuberculosis revealed by deep sequencing
Source: eLife. 2020 Feb 4;9:e53245. doi: 10.7554/eLife.53245 (PMC7012596; doi:10.7554/eLife.53245)
Supplement: Source data 1. — hSNP frequency is shown using the alignment to MT-0080_PB after removing non-MTBC reads, and filtering with the following thresholds: Phred score < 50, Root Mean Square Mapping Quality [RMS-MQ] ≤ 30, depth [DP] < 20, Read Position Rank Sum [ReadPosRankSum] < −8, Fisher Strand Bias [FS] ≥ 60 [file elife-53245-data1.docx]

**Supplementary File 1.** Percent of kmers classified as *Mycobacterium tuberculosis* Complex with hSNP frequency after removing these, reported for the alignment to MT-0080_PB and these filtering thresholds: Phred score < 50, Root Mean Square Mapping Quality [RMS-MQ] ≤ 30, depth [DP] < 20, Read Position Rank Sum [ReadPosRankSum] < -8, Fisher Strand Bias [FS] ≥ 60

| Sample | Median percent MTBC across lanes | Minimum percent MTBC across lanes | Maximum percent MTBC across lanes | Frequency of hSNPs identified after removing non-MTBC |
| --- | --- | --- | --- | --- |
| 68995 | 98.81 | 98.67 | 98.85 | 1 |
| 73787 | 98.77 | 98.63 | 98.81 | 1 |
| 74856 | 98.75 | 98.58 | 98.8 | 0 |
| 78932 | 98.75 | 98.61 | 98.78 | 3 |
| MT-0080 | 98.80 | 98.65 | 98.84 | 1 |
| MT-0712 | 98.74 | 98.56 | 98.78 | 0 |
| MT-0718 | 98.53 | 98.37 | 98.57 | 5 |
| MT-0972 | 96.99 | 96.84 | 97.04 | 0 |
| MT-1103 | 98.57 | 98.41 | 98.62 | 2 |
| MT-1206 | 98.62 | 98.49 | 98.67 | 4 |
| MT-1212 | 98.55 | 98.41 | 98.59 | 4 |
| MT-1336 | 98.47 | 98.3 | 98.52 | 6 |
| MT-1393 | 98.66 | 98.53 | 98.7 | 2 |
| MT-1466 | 98.60 | 98.42 | 98.64 | 0 |
| MT-1549 | 98.58 | 98.44 | 98.62 | 1 |
| MT-1605 | 98.60 | 98.48 | 98.65 | 3 |
| MT-1684 | 98.47 | 98.33 | 98.52 | 0 |
| MT-1838 | 98.77 | 98.61 | 98.8 | 0 |
| MT-2151 | 98.65 | 98.53 | 98.69 | 0 |
| MT-2174 | 98.57 | 98.43 | 98.62 | 3 |
| MT-2175 | 98.56 | 98.43 | 98.6 | 2 |
| MT-2184 | 98.47 | 98.33 | 98.51 | 4 |
| MT-2356 | 98.40 | 98.22 | 98.44 | 5 |
| MT-2465 | 98.15 | 98.02 | 98.2 | 3 |
| MT-2473 | 98.56 | 98.44 | 98.6 | 2 |
| MT-2474 | 98.57 | 98.42 | 98.61 | 0 |
| MT-2665 | 97.68 | 97.28 | 97.74 | 2 |
| MT-2667 | 98.45 | 98.29 | 98.5 | 7 |
| MT-2706 ^a^ | 98.77 | 98.56 | 98.78 | 1 |
| MT-2720 | 98.53 | 98.4 | 98.58 | 11 |
| MT-2762 | 98.56 | 98.41 | 98.6 | 4 |
| MT-2769 | 98.45 | 98.32 | 98.49 | 4 |
| MT-2771 | 98.74 | 98.56 | 98.79 | 0 |
| MT-2800 | 98.70 | 98.54 | 98.75 | 2 |
| MT-289 | 98.70 | 98.52 | 98.74 | 1 |
| MT-3173 | 97.92 | 97.54 | 97.97 | 0 |
| MT-3194 | 98.60 | 98.46 | 98.64 | 2 |
| MT-3255 | 98.74 | 98.57 | 98.79 | 0 |
| MT-3271 | 98.51 | 98.34 | 98.56 | 1 |
| MT-3341 | 98.80 | 98.66 | 98.85 | 3 |
| MT-3673 | 98.82 | 98.69 | 98.85 | 0 |
| MT-3683 | 98.76 | 98.59 | 98.79 | 0 |
| MT-3787 | 98.83 | 98.69 | 98.86 | 0 |
| MT-389 | 98.59 | 98.44 | 98.63 | 1 |
| MT-405 | 98.75 | 98.57 | 98.78 | 0 |
| MT-4166 | 98.50 | 98.34 | 98.54 | 5 |
| MT-467 | 98.72 | 98.55 | 98.76 | 0 |
| MT-4854 | 98.59 | 98.4 | 98.63 | 3 |
| MT-4942 | 98.57 | 98.43 | 98.62 | 6 |
| MT-504 | 98.62 | 98.5 | 98.67 | 6 |
| MT-5195 | 98.54 | 98.38 | 98.6 | 2 |
| MT-5383 | 98.72 | 98.54 | 98.76 | 1 |
| MT-5488 | 98.76 | 98.62 | 98.79 | 0 |
| MT-5531 | 98.59 | 98.4 | 98.63 | 0 |
| MT-5543 | 98.52 | 98.34 | 98.57 | 2 |
| MT-567 | 98.70 | 98.54 | 98.74 | 2 |
| MT-578 | 98.79 | 98.68 | 98.84 | 0 |
| MT-5983 | 98.76 | 98.59 | 98.81 | 2 |
| MT-6084 | 98.51 | 98.37 | 98.56 | 3 |
| MT-6218 | 98.00 | 97.69 | 98.06 | 0 |
| MT-6226 | 98.65 | 98.45 | 98.7 | 0 |
| MT-6429 | 98.63 | 98.46 | 98.67 | 2 |

^a^ Only three lanes available for this sample.
