# Supplementary material for: Previously undetected super-spreading of Mycobacterium tuberculosis revealed by deep sequencing
Source: eLife. 2020 Feb 4;9:e53245. doi: 10.7554/eLife.53245 (PMC7012596; doi:10.7554/eLife.53245)
Supplement: Source data 2. — Initial filtering thresholds used were: Phred score < 50, Root Mean Square Mapping Quality [RMS-MQ] ≤ 30, depth [DP] < 20, Read Position Rank Sum [ReadPosRankSum] < −8, Fisher Strand Bias [FS] ≥ 60. As a consequence of the depth of coverage, where allelic fraction was 0·05 < alternative allele [ALT] < 0·95, all hSNPs had at least 2 REF and ALT alleles by default. This includes all hSNPs and cSNPs identified across all samples, except variants in PE_PGRS and PPE genes, as well as those in mobile elements; some of these variants will be in positions that are excluded from the core alignment, as they failed quality control or are missing in at least one sample in the dataset. Read Position Rank Sum can only be calculated when both reference and alternative alleles are present at a position, therefore the number of cSNPs included in the summary statistics for this variable are 14720 for the H37Rv alignment and 153 for the alignment to MT-0080. *As samples were downsampled to this threshold, this is truncated at 1500. [file elife-53245-data2.docx]

**Supplementary File 2**. Comparison of consensus single-nucleotide polymorphisms (cSNPs) and heterogeneous alleles (hSNPs) in all samples aligned to H37Rv versus MT-0080_PB, after initial filtering with Allelic Fraction for cSNPs ≥ **0·95** and **0·05** < hSNP < **0·95**

|  | **cSNPs in all 62 samples** | | | | **hSNPs in all 62 samples** | | | |
| --- | --- | --- | --- | --- | --- | --- | --- | --- |
|  | **H37Rv reference, n=51430** | | **MT-0080 reference, n=365** | | **H37Rv reference, n=4897** | | **MT-0080 reference, n=125** | |
|  | **Median (IQR)** | **Range** | **Median (IQR)** | **Range** | **Median (IQR)** | **Range** | **Median (IQR)** | **Range** |
| Phred | 24922·77 (12084·77, 29426·77) | **596·85,** 55420·77 | 28115·77 (25306·77, 31561·77) | 792·77, 47999·77 | 3820·76 (1065·77, 8864·77) | 50·77, 37538·77 | 136·77 (84·77, 1872·77) | 50·77, 26808·77 |
| RMS-MQ | 60 (60, 60) | **39·13**, 69·42 | 60 (60, 60) | 39·75, 60 | 58·91 (56·62, 59·95) | 33·81, 65·42 | 60 (60, 60) | 46·61, 60 |
| DP | 670 (322, 783) | 20, 1468 | 758 (683, 844) | 23, 1235 | 801 (421, 1056) | 20, 1500* | 256 (78, 687) | 32, 990 |
| ReadPosRankSum | -0·064 (-1·093, 1·043) | **-5·269**, 3·735 | 010 (-0·783, 0·913) | -2·84, 2·71 | 1·383 (-2·691, 5·418) | -7·992, 15·309 | 0·892  (-0·501, 2·502) | -6·366, 4·101 |
| FS | 0 (0, 0) | 0, **45·053** | 0 (0, 0) | 0, 2·28 | 9·28 (1·979, 26·173) | 0, 59·985 | 1·723 (0·685, 4·69) | 0, 58·993 |
