# Supplementary material for: Previously undetected super-spreading of Mycobacterium tuberculosis revealed by deep sequencing
Source: eLife. 2020 Feb 4;9:e53245. doi: 10.7554/eLife.53245 (PMC7012596; doi:10.7554/eLife.53245)
Supplement: Source data 5. — Quast (v.5.0.2 Gurevich et al., 2013) was used to tabulate the above statistics, with the exception of the number of CDS and RNA, where annotation was done using RASTtk (v.2.0 Brettin et al., 2015). [file elife-53245-data5.docx]

**Supplementary File 5.** Assembly metrics for Single Molecule Real-Time sequencing of MT-0080 (‘MT-0080_PB’), aligned to NC_000962.3 (H37Rv).

|  | **MT-0080_PB** |
| --- | --- |
| Number of contigs | 1 |
| Largest contig, length in bp | 4,426,525 |
| GC content, % | 65·61 |
| Genome fraction covered, % | 99·28 |
| Largest alignment, bp | 592,812 |
| Total aligned length, bp | 4,387,983 |
| NG50 | 4,426,525 |
| NA50 | 256,105 |
| Reads mapped, % | 97·48 |
| Average depth of coverage, using filtered reads | 235 |
| Coverage ≥ 10x, % | 100 |
| Number of relocations | 50 |
| Number of inversions | 1 |
| Number of missing bases (‘N’) | 0 |
| Number of CDS | 4,321 |
| Number of RNA | 47 |
| Average nucleotide identity to H37Rv | 99·92% |
