# Supplementary material for: Previously undetected super-spreading of Mycobacterium tuberculosis revealed by deep sequencing
Source: eLife. 2020 Feb 4;9:e53245. doi: 10.7554/eLife.53245 (PMC7012596; doi:10.7554/eLife.53245)
Supplement: Source data 9. — Initial filtering thresholds used were: Phred score < 50, Root Mean Square Mapping Quality [RMS-MQ] ≤ 30, depth [DP] < 20, Read Position Rank Sum [ReadPosRankSum] < −8, Fisher Strand Bias [FS] ≥ 60. Where 0·01 < alternative allele [ALT] < 0·99, a minimum of 2 REF/ALT alleles were required for all hSNPs to reduce risk of including sequencing error; those that failed to meet these criteria were excluded. This includes all hSNPs and cSNPs identified across all samples, except variants in PE_PGRS and PPE genes, as well as those in mobile elements; some of these variants will be in positions that are excluded from the core alignment, as they failed quality control or are missing in at least one sample in the dataset. Read Position Rank Sum can only be calculated when both reference and alternative alleles are present at a position, therefore the number of cSNPs included in the summary statistics for this variable are 13255 for the H37Rv alignment and 148 for the alignment to MT-0080 in the cSNPs ≥ 0·99 and 0·01 < ALT < 0·99 analysis. *As samples were downsampled to this threshold, this is truncated at 1500. P values were calculated using on the Wilcoxon-Mann-Whitney test. [file elife-53245-data9.docx]

**Supplementary File 9**. Comparison of consensus single-nucleotide polymorphisms (cSNPs) and heterogeneous alleles (hSNPs) in all samples aligned to H37Rv versus MT-0080_PB, after initial filtering with Allelic Fraction for cSNPs ≥ **0·99** and **0·01** < hSNP < **0·99**

|  | **cSNPs in all 62 samples** | | | | **hSNPs in all 62 samples** | | | |
| --- | --- | --- | --- | --- | --- | --- | --- | --- |
|  | **H37Rv reference, n=49965** | | **MT-0080 reference, n=361** | | **H37Rv reference, n=5819** | | **MT-0080 reference, n=129** | |
|  | **Median (IQR)** | **Range** | **Median (IQR)** | **Range** | **Median (IQR)** | **Range** | **Median (IQR)** | **Range** |
| Phred | 25180·77 (15127·77, 29568·77) | 610·77, 55420·77 | 28133·77 (25313·77, 31561·77) | 792·77, 47999·77 | 4457·77 (1338·53, 8490·77) | 50·77, 37538·77 | 157·77 (84·77, 1986·77) | 50·77, 43450·77 |
| RMS-MQ | 60 (60, 60) | 39·13, 69·31 | 60 (60, 60) | 39·75, 60 | 59·41 (56·64, 60) | 33·81, 69·25 | 60 (60, 60) | 46·61, 60 |
| DP | 676 (415, 787) | 20, 1468 | 759 (683, 844) | 23, 1235 | 664 (233, 1017) | 20, 1500* | 271 (79, 691) | 32, 1191 |
| ReadPosRankSum | 0·032 (-0·975, 1·09) | -2·898, 2·82 | 0·118 (-0·738, 0·953) | -1·868, 2·71 | 0·628 (-2·273, 4·538) | -7·992, 15·309 | 0·773 (-0·624, 2·475) | -6·366, 4·101 |
| FS | 0 (0, 0) | 0, 9·514 | 0 (0, 0) | 0, 0 | 5·933 (1·018, 22·791) | 0, 59·985 | 1·623 (0, 4·676) | 0, 58·993 |
